# Stereological Evidence of Non-Selective Hippocampal Neurodegeneration, IGF-1 Depletion, and Behavioral Deficit following Short Term Bilateral Adrenalectomy in Wistar Rats

**DOI:** 10.3390/biom13010022

**Published:** 2022-12-22

**Authors:** Naserddine Hamadi, Ömür Gülsüm Deniz, Ahlam Said Abi Issa, Azim Ullah Shamsul Islam, Naheed Amir, Saeed Tariq Minhas, Nather Madjid, Fatima Khelifi-Touhami, Süleyman Kaplan, Abdu Adem

**Affiliations:** 1 Department of Life and Environmental Sciences, College of Natural and Health Sciences, Zayed University, Abu Dhabi P.O. Box 144534, United Arab Emirates; 2 Department of Histology and Embryology, Medical Faculty, Bolu Abant Izzet Baysal University, Bolu 14030, Turkey; 3 Department of Pharmacology and Therapeutics, College of Medicine and Health Sciences, United Arab Emirates University, Al Ain P.O. Box 17666, United Arab Emirates; 4 Department of Anatomy, College of Medicine and Health Sciences, United Arab Emirates University, Al Ain P.O. Box 17666, United Arab Emirates; 5 Department of Neuroscience, Karolinska Institutet, SE-171 77 Stockholm, Sweden; 6 Department of Animal Biology, Faculty of Life and Science, Constantine University-1, Ain Elbey Street, Constantine 2500, Algeria; 7 Department of Histology and Embryology, Medical Faculty, Ondokuz Mayıs University, Samsun 55139, Turkey; 8 Department of Pharmacology and Therapeutics, College of Medicine and Health Sciences, Khalifa University, Abu Dhabi P.O. Box 127788, United Arab Emirates

**Keywords:** hippocampus, neurodegeneration, adrenalectomy, passive avoidance, stereology

## Abstract

The development of animal models to study cell death in the brain is a delicate task. One of the models, that was discovered in the late eighties, is the induction of neurodegeneration through glucocorticoid withdrawal by adrenalectomy in albino rats. Such a model is one of the few noninvasive models for studying neurodegeneration. In the present study, using stereological technique and ultrastructural examination, we aimed to investigate the impact of short-term adrenalectomy (2 weeks) on different hippocampal neuronal populations in Wistar rats. In addition, the underlying mechanism(s) of degeneration in these neurons were investigated by measuring the levels of insulin-like growth factor-1 (IGF-1) and β-nerve growth factor (β-NGF). Moreover, we examined whether the biochemical and histological changes in the hippocampus, after short-term adrenalectomy, have an impact on the cognitive behavior of Wistar rats. Stereological counting in the hippocampus revealed significant neuronal deaths in the dentate gyrus and CA4/CA3, but not in the CA2 and CA1 areas, 7 and 14 days post adrenalectomy. The ultrastructural examinations revealed degenerated and degenerating neurons in the dentate, as well as CA4, and CA3 areas, over the course of 3, 7 and 14 days. The levels of IGF-1 were significantly decreased in the hippocampus of ADX rats 24 h post adrenalectomy, and lasted over the course of two weeks. However, β-NGF was not affected in rats. Using a passive avoidance task, we found a cognitive deficit in the ADX compared to the SHAM operated rats over time (3, 7, and 14 days). In conclusion, both granule and pyramidal cells were degenerated in the hippocampus following short-term adrenalectomy. The early depletion of IGF-1 might play a role in hippocampal neuronal degeneration. Consequently, the loss of the hippocampal neurons after adrenalectomy leads to cognitive deficits.

## 1. Introduction

In the late eighties, Sloviter et al. [1] showed a high dependence of granule cells in the hippocampus of Long Evans rats on glucocorticoids for their survival. They discovered that the withdrawal of glucocorticoids by adrenalectomy triggers a massive granule cell loss, and spares the degeneration of pyramidal cells [1]. This is an important and useful model of selective, experimentally controlled, neuronal cell death in the hippocampus. It is important to understand the different factors involved in neurodegeneration which might be relevant to neurodegenerative disorders, such as Alzheimer’s disease where a region such as the hippocampus is primarily affected.

Several other groups replicated the findings [2,3,4,5]. However, Sapolsky et al. [6] were the first to show that in addition to the degeneration of hippocampal granule cells, a significant decrease in the number of CA4 pyramidal cells took place following the adrenalectomy. Our previous study [7] supported and extended the findings of Sapolsky et al. [6]. We found, in our previous studies, using cell counting and electron microscopy, that long-term adrenalectomy (5 months) of Wistar rats caused pyramidal cell death, not only in the CA4 but in different areas of the Cornu Ammonis CA3, CA2, and CA1 alongside a drastic and extensive degeneration in the granule cells of the dentate gyrus [7,8]. Furthermore, Martinez-Carlos et al. [9] demonstrated apical dendritic atrophy in the CA3 region of the hippocampus following adrenalectomy (35 days). It is worth noting that following short- and long-term adrenalectomy, the only affected region in the brain is the hippocampus [1,2,6].

The survival of neurons depends on different growth hormones such as insulin-like growth factors (IGFs) and Beta-Nerve growth factor (β-NGF). IGF-1 is a critical neurotrophic protein essential for optimal brain development, cell survival, and growth [10]. It is produced on a cellular level in various tissues, including the brain, where it performs paracrine functions [11]. The major CNS cell types in the hypothalamus, hippocampus, cerebellum, and cortex produce IGF-1 [12,13,14,15].

IGFs are polypeptides with a sequence similar to insulin. IGF-I and IGF-II receptors are abundantly expressed in the brain where IGF signaling mediates nervous system maintenance, myelination, and synapse formation [16]. These roles of the IGFs are attributed to their ability to cross the blood-brain barrier, and their endocrine roles in the brain [17]. Furthermore, a number of studies have reported the involvement of IGFs in the brain’s cognitive functions [18]. In addition, β-NGF is a polypeptide found in the central nervous system (CNS) in which the hippocampus contains the highest levels of this hormone [19]. β-NGF is an essential neurotrophic factor in the developing sympathetic and sensory nervous systems [20]. It exerts a number of different effects on neurons, such as neurogenesis [21], neuronal plasticity [22], development [23], and differentiation [24]. Neurons that fail to obtain sufficient β-NGF die by apoptosis [25]. It has been shown that short-term adrenalectomy in young rats caused a drastic decrease in the β-NGF levels in the hippocampus [26]. Glucocorticoids, IGF-1 and β-NGF, play important roles in the brain; nonetheless, their interactions remain unclear although some studies have suggested a relation between them [27,28,29]. Furthermore, any disturbance in the levels of such hormones directly affects the survival of neurons, and this might be reflected in the cognitive capability of the animals. The hippocampus is known for its major role in learning and memory, and several investigations, including our own, indicated a decline in memory performance tasks and learning in long-term ADX rats, highlighting the possibility of the involvement of neuronal damage [7,30,31,32].

One of the purposes for conducting the present study was to investigate the effect of short-term adrenalectomy on neuronal cell death in the hippocampus. Therefore, we investigated cell death in the hippocampus after short-term adrenalectomy, and explored whether both granule and pyramidal cells were vulnerable to the absence of glucocorticoids. In addition, we aimed to explore the impact of short-term adrenalectomy on the levels of two main growth factors IGF-1 and β-NGF. Moreover, we examined whether neuronal death after short-term ADX in the hippocampus of different populations has an impact on the cognitive behavior of the animals.

## 2. Materials and Methods

### 2.1. Animals and Adrenalectomy

Eight-week-old male albino Wistar rats 170–220 g were obtained from the College of Medicine and Health Sciences animal facility (Al Ain, United Arab Emirates), and used in the current study. We have used 80 rats for biochemical analysis, 50 rats for stereology, 18 rats for electron microscopy examination, and 56 rats for the Passive Avoidance (PA) task. Under pentobarbital (35 mg/kg body weight, Ilium-Troy Laboratories, New South Wales, Australia) anesthesia, rats were subjected either to adrenalectomy or to SHAM operation (laparotomy), as described by Sloviter et al. [1]. Shaving the backs of the animals was carried out using an electric shaving machine (Wahl, Sterling, IL, USA). Adrenalectomy and SHAM surgery were performed between 9:00 a.m. to 13:00 p.m. The rat was placed on the surgical table ventrally, and in order to perform an aseptic surgery, the back of the animal was cleaned with 70% ethanol (Sigma-Aldrich, City of Saint Louis, MO, USA). Bilateral incisions were made into the skin and the dorsal muscle to access the peritoneal cavity. The size of the muscle incision was just big enough to expose the adrenal gland on the top of the kidney, and provided some free space to remove it without leaving any intact residuals of the gland. The periadrenal fat was grasped using blunt forceps, and the adrenal gland was exteriorized with tipped-blunt scissors. A cut was made at the connective tissue between the kidney and the adrenal gland. The incision was sutured, and the animal was returned to the cage. The same procedure was applied to the SHAM operated animals, except for the removal of the adrenal gland.

**Figure 1:**
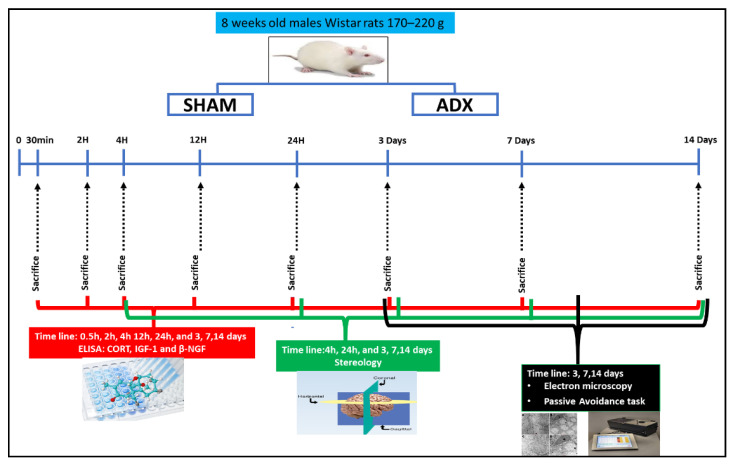
Schematic illustration of adrenalectomy (surgery), biochemical measurements of corticosterone (CORT), insulin-like growth factor-1 (IGF-1) and β-nerve growth factor (β-NGF), stereology cell counting, electron microscopy and Passive Avoidance task. The levels of corticosterone (CORT) in the serum were measured by Enzyme Immunoassay (EIA) to assess the efficacy of adrenalectomy (n = 204) over the course of time (30 min, 2 h, 4 h, 24 h and 3, 7, and 14 days). Using ELISA, we measured the levels of IGF-1 and β-NGF in the hippocampal homogenates of ADX and SHAM operated rats over the course of time 30 min (SHAM = 5, ADX = 5), 2 h (SHAM = 5, ADX = 5), 4 h (SHAM = 5, ADX = 5), 12 h (SHAM = 5, ADX = 5), 24 h (SHAM = 5, ADX = 5), 3 days (SHAM = 5, ADX = 5), 7 days (SHAM = 5, ADX = 5) and 14 days (SHAM = 5, ADX = 5). The quantification of pyramidal and granule cells was performed by stereological analysis on the hippocampus at multiple time points 4 h (SHAM = 5, ADX = 5), 24 h (SHAM = 5, ADX = 5), 3 days (SHAM = 5, ADX = 5), 7 days (SHAM = 5, ADX = 5) and 14 days (SHAM = 5, ADX = 5). Electron microscopy was used to examine the thin sections of the hippocampi of ADX rats and SHAM operated over the course of 3 days (SHAM = 3, ADX = 3), 7 days (SHAM = 3, ADX = 3) and 14 days (SHAM = 3, ADX = 3). The Passive Avoidance (PA) task was used to examine the cognitive function over the course time 3 days (SHAM = 6, ADX = 9), 7 days (SHAM = 8, ADX = 10) and 14 days (SHAM = 7, ADX = 16).

All animals were placed in plexiglas cages, four rats per group, where a temperature of 22 °C and 60% humidity were maintained. Rats were housed in a 12:12 h normal light–dark cycle, and received ad libitum access to food and water throughout the experiment. ADX rats were provided with 0.9% saline instead of drinking water, in order to maintain electrolyte balance and prevent the deleterious effects of sodium chloride insufficiency. Subsequently, randomly selected rats from each group were sacrificed for biochemical analysis, stereology, and electron microscopy. For the behavioral test, a separate set of animals were selected to assess the cognitive deficit, as it is indicated in Figure 1.

### 2.2. Determination of Serum Corticosterone Levels

The level of corticosterone (CORT) in the serum was used to assess the efficacy of adrenalectomy. Approximately 1 mL blood samples were taken directly from the heart at the time of sacrifice by head decapitation using a guillotine. The sera samples were stored at −80 °C until CORT levels were measured by Enzyme Immunoassay (EIA) Kit (Life Sciences, Lausanne, Switzerland) according to the manufacturer’s protocol. The procedure uses a polyclonal antibody to CORT to bind in a competitive manner. A standard curve in the range from 32 to 20,000 pg/mL was constructed to calculate CORT concentrations in the samples.

### 2.3. Stereological Analysis

Stereological quantification of pyramidal and granule cells was performed on the hippocampus as described earlier [33]. The total number of animals used was 50 animals, distributed as follows: 4 h (SHAM = 5, ADX = 5), 24 h (SHAM = 5, ADX = 5), 3 days (SHAM = 5, ADX = 5), 7 days (SHAM = 5, ADX = 5) and 14 days (SHAM = 5, ADX = 5). Briefly, three series of tissue sections were processed for cresyl fast violet (Nissl) staining to label neuronal cells. Stereological analysis was performed with a stereology workstation (Computer Assisted Stereological Toolbox (CAST) grid, Olympus, Denmark). Slides to count were determined in systematic random sampling (SRS), as described earlier [30]. The total number of pyramidal and granule cells was estimated using the optical dissector method. Counts included all neuronal cells distributed in a systematic random fashion within an unbiased counting frame throughout the hippocampus. Cell counting in the hippocampus was performed blindly by the investigator. SRS determined the selection of sampled areas in each section; this approach does not allow a researcher to select the sampled area, and give an equal chance to the whole tissue to be sampled. After completing cell number estimation (pyramidal and granular cells), data were decoded, and statistical analysis was conducted.

### 2.4. Transmission Electron Microscopy

At each designated time point (3, 7, and 14 days), animals (n = 18) were deeply anesthetized with intraperitoneal administration of sodium pentobarbital (35 mg/kg body weight). Transcardial perfusion-fixation was performed through the ascending aorta, with 50 mL of 0.1 M phosphate buffered saline, followed by 300 mL of freshly prepared solution of Karnovsky’s fixative, pH 7.2, at 3 days (n = 6), 7 days (n = 6), and 14 days (n = 6) after the surgery. For electron microscopy, samples were immersed in McDowell and Trump fixative for 3 h at 25 °C as described previously [34]. Tissues were rinsed with phosphate buffer saline, and fixed with 1% osmium tetroxide for 1 h. Samples were washed with distilled water, dehydrated in graded ethanol, and propylene oxide. Finally, the tissue was embedded in Agar-100 epoxy resin at 65 °C for 24 h. Blocks were trimmed and semi-thin and ultra-thin sections were cut with Reichert Ultracuts (ultramicrotome), approximately −3.7 mm relative to bregma, according to Paxinos and Watson. [35]. The ultrathin sections (95 nm) mounted on 200 mesh Cu grids were contrasted with uranyl acetate, followed by lead citrate double stain. The sections on the grids were examined by an electron microscope specialist that was not aware of the experiment that we were conducting, in addition to the grouping of the animals. Sections were photographed under a Philips CM10 transmission electron microscope [34].

### 2.5. Enzyme-Linked Immunosorbent Assay (ELISA)

We used ELISA to evaluate the levels of two growth factors (IGF-1 and β-NGF) in the hippocampal homogenates of ADX and SHAM operated rats. Eighty rats were used as follows: 30 min (SHAM = 6, ADX = 6), 2 h (SHAM = 6, ADX = 6), 4 h (SHAM = 6, ADX = 6), 12 h (SHAM = 6, ADX = 6), 24 h (SHAM = 5, ADX = 5), 3 days (SHAM = 5, ADX = 5), 7 days (SHAM = 5, ADX = 5), and 14 days (SHAM = 5, ADX = 5).

IGF-1, a trophic factor for neurons, has also been shown to be an important regulator of cell metabolism, differentiation, and survival. The Quantikine^®^ ELISA from R&D Systems^®^ assay was used for the quantitative determination of rat IGF-1 levels in tissue homogenates of the hippocampi, as described in the manufacturer’s protocol. A monoclonal antibody specific for rat IGF-I had been pre-coated onto a microplate. Any rat IGF-I present in the sample was bound by the immobilized antibody. An enzyme-linked polyclonal antibody specific for rat IGF-I was added to the wells. Following the formation of bound antibody enzyme reagent, a substrate solution yielded a blue product that turned yellow with the addition of the Stop Solution. The intensity of the color measured at 450 nm was in proportion to the amount of rat IGF-I bound in the initial step.

β-NGF is a trophic factor for neurons, and is involved in the maintenance of the sympathetic and sensory nervous systems. A DuoSet^®^ ELISA from R&D Systems^®^ assay was used for the quantitative measurement of rat β-NGF levels in hippocampal tissue homogenate, as described in the manufacturer’s protocol. We used a specific antibody for rat β-NGF, goat anti rat β-NGF, coated on a 96 well plate. The β-NGF present in a sample was bound to the wells by the immobilized antibody. Later, biotinylated goat anti rat β-NGF antibody was added, followed by HRP conjugated streptavidin. The addition of the substrate solution (1:1 tetramethylbenzidine and H_2_O_2_) allowed color development in proportion to the amount of β-NGF bound. Finally, the Stop Solution (2N sulfuric acid; H_2_SO_4_) changed the color from blue to yellow. The intensity of the color was measured at 450 nm, which was in proportion to the concentration of β-NGF in the hippocampal homogenate.

### 2.6. Step-through Passive Avoidance Test

In order to examine the impact of the aforementioned biochemical and cellular alterations in the hippocampus on cognitive function, the Passive Avoidance (PA) task was used, as described earlier [36,37]. Fifty-six animals were used as follows: 3 days (SHAM = 6, ADX = 9), 7 days (SHAM = 8, ADX = 10), and 14 days (SHAM = 7, ADX = 16). All animals were handled daily for at least 3 days before the experiments, between 9:00 am to 15:00 pm. The PA task was conducted between 9:00 am to 15:00 pm using a two-compartment standard shuttle box (51 × 25 × 24 cm) (Harvard Apparatus, Holliston, MA, USA). The two compartments were of equal size and had a stainless-steel bar floor connected by a built-in sliding door (7 × 7 cm). The right-hand compartment (shock compartment) was painted black to obtain a dark chamber, while the left hand compartment was illuminated by a bulb (24 V; 5 W) installed on the top Plexiglas cover. PA training was conducted in a single session (day 1).

The rats were placed in the lighted compartment (with no access to the dark compartment) and were allowed to explore for 60 s. During the exploration period in the PA apparatus, when 60 s expired, the sliding door was automatically opened, and the rat was allowed to cross over into the dark compartment. Once the rat had entered the dark compartment with all four paws, the sliding door was automatically closed and a weak electrical current (constant current, scrambled, duration 3 s, 0.3 mA) was delivered through the grid floor. Latency to cross into the dark compartment (training latency) was recorded. If a rat failed to move into the dark compartment within 300 s (cut off latency), the door was reopened and the rat was gently moved into the dark compartment, where it received the foot shock. After exposure to the foot shock, the rat was allowed to stay for 30 s in the dark compartment before it was removed from the PA apparatus, and returned to its home cage.

Retention was tested 24 h after training (day 2) [36,37]. The animal was again placed in the lighted (safe) compartment, with access to the dark compartment for a period of 300 s. The latency to enter the dark compartment with all four paws was automatically measured (retention latency). If the rat failed to enter the dark compartment within 300 s, it was removed and assigned a maximum test latency score of 300 s.

### 2.7. Statistical Analysis

All data are reported as the mean ± standard error of the mean (±SEM) and the analysis was considered significantly different if *p* ≤ 0.05. Using SPSS version 28 (IBM, Armonk, NY, USA), the data were evaluated by a two-way ANOVA method and Bonferroni test for growth factors. A two-tail Student’s *t*-test was used to analyze the stereology and Passive Avoidance data. The normality of the data was verified using a one sample Kolmogorov–Smirnov Test.

## 3. Results

### 3.1. Determination of Serum Corticosterone Levels

**Figure 2:**
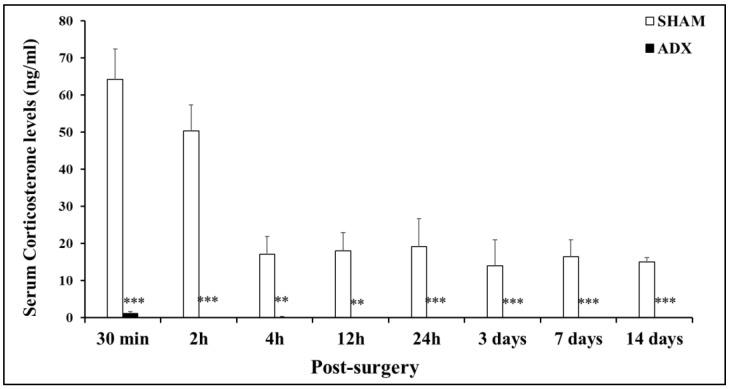
Bar graph showing the levels of serum corticosterone (CORT). Levels of CORT in the serum of ADX rats and SHAM operated rats were measured by Enzyme Immunoassay (EIA) to assess the efficacy of adrenalectomy (n = 204) over the course of time (30 min, 2 h, 4 h, 12 h, 24 h and 3, 7, and 14 days). ** *p* < 0.01 and *** *p* < 0.001. Data are expressed as mean (±SEM).

Our results showed that the removal of the adrenal gland caused a significant decrease in the levels of corticosterone at 30 min (*p* < 0.001), 2 h (*p* < 0.001), and 4 h (*p* < 0.01) in ADX rats compared to SHAM operated. The levels of corticosterone were undetectable in the serum of ADX rats at 12 h, 24 h, and 3, 7, and 14 days compared to the SHAM operated rats (Figure 2).

A significant effect of post-surgical time was present for SHAM operated rats. Means of the CORT levels were 64.17 ± 8.26, 50.34 ± 7.04, 17.07 ± 4.81, 18.02 ± 4.93, 19.14 ± 7.52, 13.95 ± 7.03, 16.45 ± 4.60, and 15.00 ± 1.18 ng/mL at the 30 min, 2 h, 4 h, 12 h, 1 day, 3 days, 7 days, and 14 days’ time points, respectively.

### 3.2. Stereological Analysis

Our stereological results showed that the effect of adrenalectomy on the hippocampus is not selective. The removal of the adrenal glands leads to the reduction in the levels of glucocorticoids to undetectable levels, ultimately causing the death of different types of neuronal cells throughout the hippocampus.

**Figure 3:**
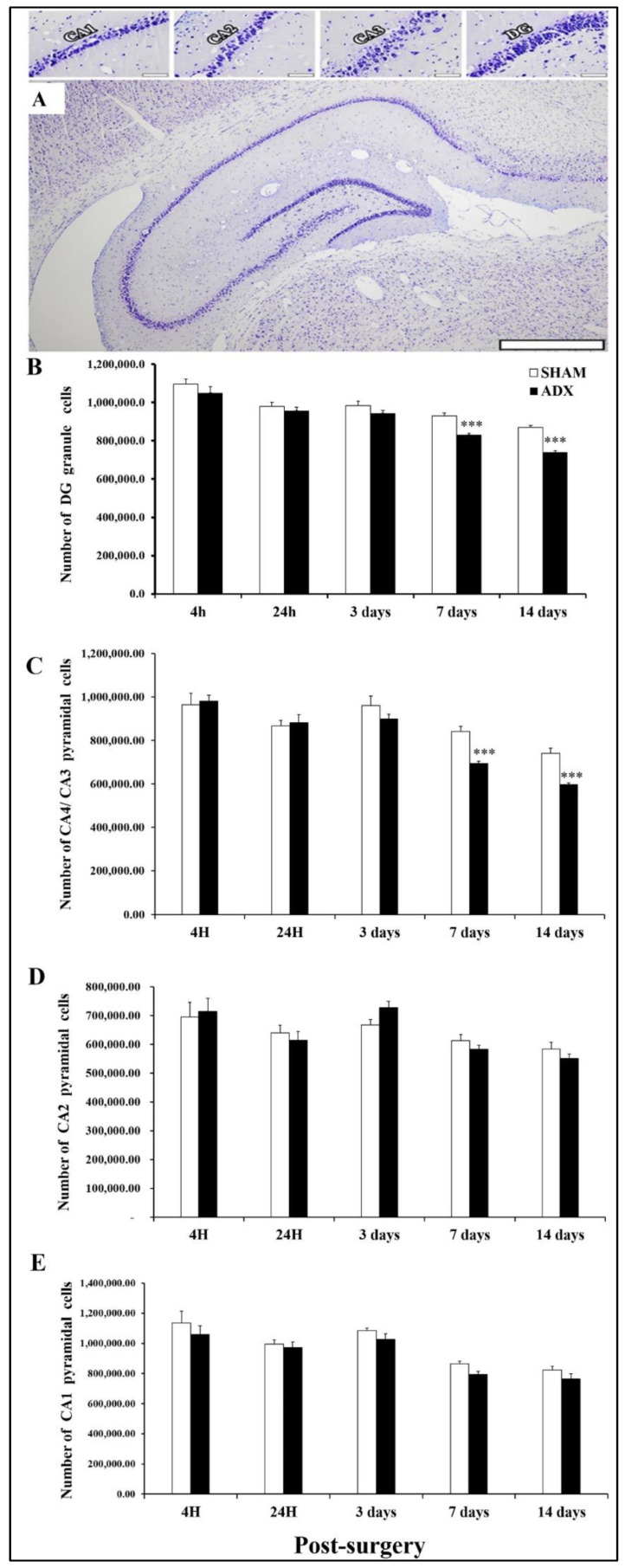
Stereological evidence of non-selective cell death in the hippocampus following adrenalectomy over the course of (4 h, 24 h and 3, 7, 14 days). (**A**) A general view of hippocampus and cell counting in the different regions of the CA1, CA2, CA3 and DG were investigated. This is a representative image of the hippocampus. Estimating total neuron number by stereological technique was performed on more than 20 sections and one hundred areas in each subject and region. Notice that images taken from one section may not represent the results gained by counting hundreds of sampled cells in each section. Since the orientation of section cutting may result in a very different sectioned surface area for each sampled area, for this reason, substantial neuron loss may not be seen in the image. Scale bar = 50 µm in CA1, CA2, CA3 and DG images. Scale bar = 500 µm in the full hippocampus image. (**B**) showing the number of DG granule cells in the hippocampus of ADX and sham operated rats. No difference in the number of neurons was observed 3 days postoperatively. However, a significant decrease in the number of granule cells was observed 7 and 14 days following adrenalectomy in ADX rats, compared to sham operated. (**C**) showing the number of CA4/CA3 neurons in the hippocampus of adrenalectomized and sham operated rats over the course (4 h, 24 h, 3 days, 7 days, and 14 days). No difference in the number of CA4/CA3 neurons up to 3 days, but a significant decrease was observed 7 and 14 days following adrenalectomy. (**D**) Bar graphs showing the number of CA2 neurons in the hippocampus of ADX and sham operated rats over the course of 4 h, 24 h, 3 days, 7 days, and 14 days. No difference in the numbers of neurons following adrenalectomy. (**E**) showing the number of CA1 neurons in the hippocampus of ADX and sham operated rats. No difference in the number of neurons was revealed over the course of time (4 h, 24 h, 3 days, 7 days, and 14 days) in both ADX and sham operated rats. *** *p* < 0.001. Data are expressed as mean (±SEM).

The removal of the adrenal glands is deleterious to the neurons in the dentate gyrus of the hippocampus of ADX, compared to sham operated rats, starting from the third day postoperatively. As shown in Figure 3B, in the first three days following adrenalectomy the numbers of cells in the DG were affected, but it did not reach a statistical significance. However, after 7 days, postoperatively, the number of cells in the dentate gyrus decreased significantly (*p* < 0.001), and such an effect continued to be seen 14 days later in the ADX rats compared to the sham operated rats (*p* < 0.001).

Our stereology results showed that the removal of the adrenal glands affected the survival of the CA4 and CA3 pyramidal cells in the hippocampus. We observed significant loss (*p* < 0.001) of CA4 and CA3 neurons 7 and 14 days post adrenalectomy in the ADX rats compared to sham operated rats (Figure 3C).

In contrast, our data showed that the number of pyramidal cells in the CA2 was not affected significantly over the course of time (4 h, 24 h, 3 days, 7 days, and 14 days) in both ADX and sham operated rats (Figure 3D).

Similar to what we have seen with the pyramidal cells of CA2, the removal of the adrenal gland did not affect the neuronal population of the CA1 area (Figure 3E).

### 3.3. Transmission Electron Microscopy

In the current study, we aimed to examine the impact of short-term adrenalectomy on the ultrastructure of the different cells of the hippocampus at different time points (3, 7, and 14 days).

**Figure 4:**
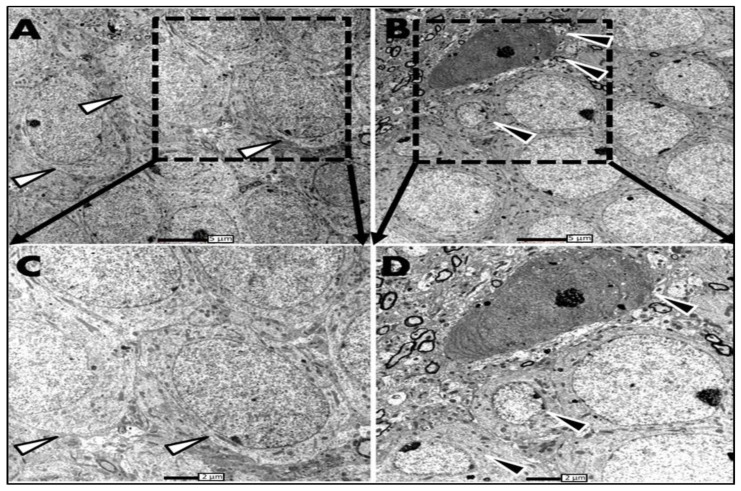
Electron micrograph of the upper blade of the dentate gyrus, three days postoperatively, of ADX rats compared to SHAM operated. (**A**) In the hippocampus SHAM rats granule cells of the dentate gyrus have a round shape and a big nucleus occupies the whole cell body; in such cells, the nucleus is surrounded by a thin layer of cytoplasm (white arrows). (**B**) in the ADX rats a few degenerated granule cells were seen on the tip of the upper blade where deformation of the cell morphology was seen, in addition to the shrinkage of the cell body. The darkly stained cell is going to die; a few days later it will be removed from the region (black arrow). Scale bar = 5 µm. (**C**,**D**) higher magnification of granule layer of dentate gyrus of SHAM operated and ADX rats, respectively. Scale bar = 2 µm. Three independent sections per brain were examined (n = 3 in each group).

The examination of thin sections of the hippocampi of SHAM operated and ADX rats under the electron microscope, three days postoperatively, showed granule cell degeneration on the tip of the upper blade of the dentate gyrus where the cells exhibited condensed chromatin, an irregular cell membrane, and the beginning of vacuolation in the cytoplasm; however, this was only the case in ADX rats (Figure 4B,D) compared to the sham operated (Figure 4A,C). Interestingly, we have not observed any cell abnormalities in the rest of the dentate gyri of the ADX rats (Figure 4B,D).

**Figure 5:**
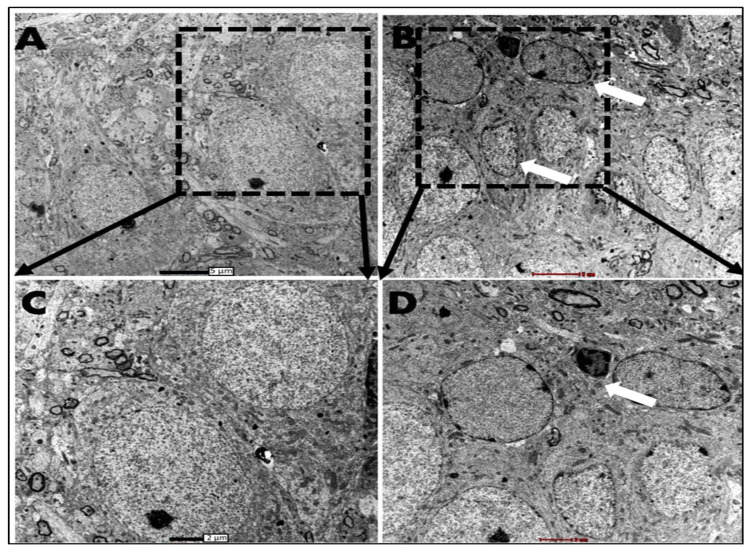
Electron micrographs of CA4 pyramidal cells, three days postoperatively, of ADX rats compared to SHAM operated. (**A**) Healthy pyramidal cells in the hippocampus of SHAM rats showing well defined nucleolus and nucleus and homogenous karyoplasm. (**B**) multiple degenerated pyramidal neurons of the CA4 in ADX rats with irregular nucleus membrane and signs of chromatin condensation; the arrow showing already degenerated neuron where reduction of the nucleus volume is apparent and dark compacted chromatin. Scale bar = 5 µm. (**C**,**D**) higher magnification of pyramidal layer of CA4 of SHAM operated, compared to ADX rats, respectively. Scale bar = 2 µm. Three independent sections per brain were examined (n = 3 in each group).

Concerning the different areas of the Cornu Ammonis (CA areas) three days post adrenalectomy, we found that solely CA4 pyramidal cells displayed signs of cell degeneration (Figure 5B,D), compared to SHAM operated rats (Figure 5A,C). Nevertheless, pyramidal cells of CA3, CA2, and CA1 cells did not show any signs of neurodegeneration after three days of surgery in both groups (results are not shown).

**Figure 6:**
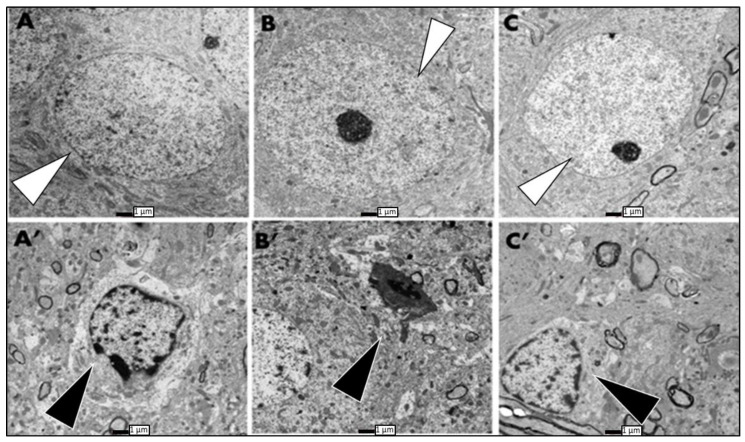
Electron micrographs showing degeneration in different areas of the hippocampus of ADX rats compared to the SHAM operated; these were taken seven days postoperatively. (**A**) intact granule cell with well-defined nucleus and cell membrane of SHAM rats (white arrows). (**A′**) degenerated granule cell of the dentate gyrus with condensed chromatin, and irregular cell and nuclear membrane in ADX rats (black arrow). (**B**,**C**) show healthy pyramidal cells of CA4 and CA3, respectively, in SHAM rats (white arrows). (**B′**) showing ultrastructure of CA4 dead neurons in ADX rats with disintegration of the cell body, condensation of the chromatin, and reduction of the nucleus volume (black arrow). (**C′**) degenerated pyramidal neurons of the CA3 in ADX rats showing irregular nucleus membrane and chromatin condensation (black arrow). Scale bar = 1 µm. Three independent sections per brain were examined (n = 3 in each group).

Seven days following adrenalectomy, the ultrastructural examination of the hippocampus revealed a progression in neurodegeneration, compared to the third day of adrenalectomy where cell death was seen all over in both blades of the dentate gyrus, and more extensively on the tip of the upper blade where we observed more exacerbation of the neurodegenerative process (Figure 6A′). In addition to what we have seen on the third day after adrenalectomy, more degenerative cells were observed in the CA4 seven days postoperatively (Figure 6B′). Electron microscopy examination, of the neuropil in the CA3 area of the hippocampus of ADX rats, revealed cell death of the pyramidal cells for the first time seven days following adrenalectomy (Figure 6C′). No sign of degeneration was seen in the CA1 and CA2 of the adrenalectomized rats. The hippocampus of the SHAM operated rats did not show any abnormalities in different neuronal populations (Figure 6A–C).

**Figure 7:**
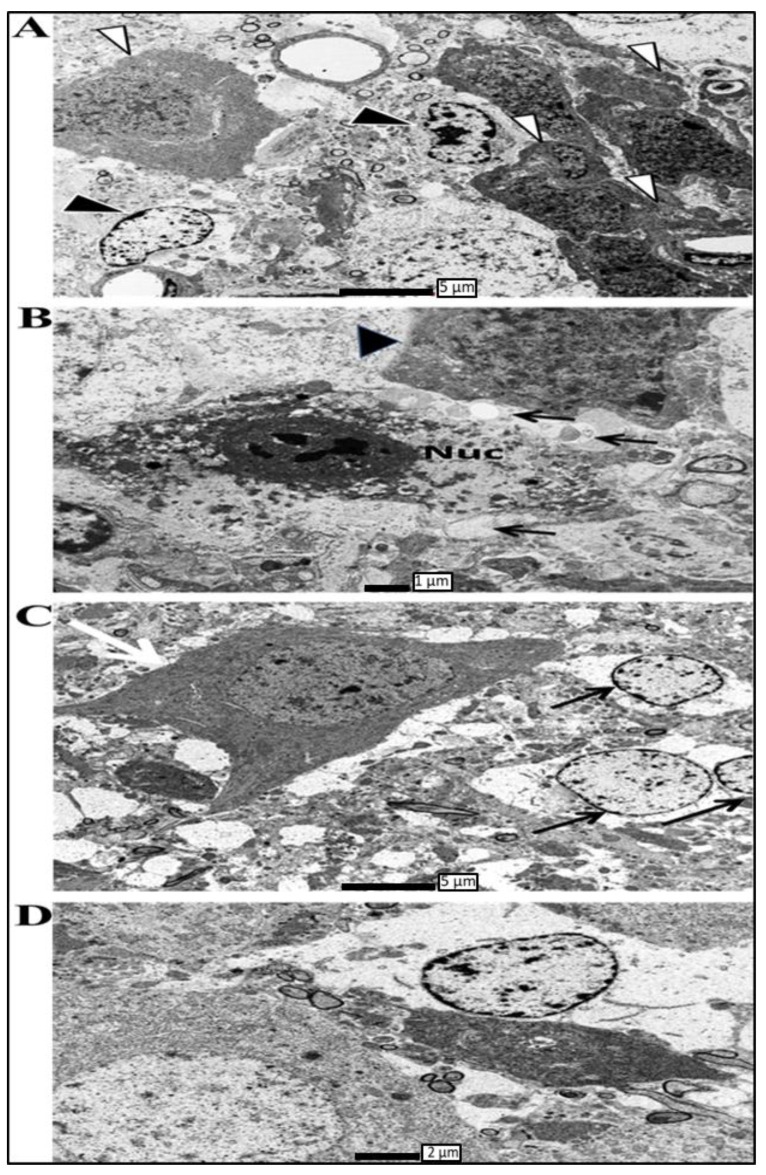
Electron micrographs showing degeneration in different areas of the hippocampus of ADX rats, compared to the SHAM operated, were taken 14 days postoperatively. (**A**) Electron micrographs taken from the upper blade of the dentate gyrus 14 days following adrenalectomy showing granule cell engulfed by microglia (white arrows); in addition, it shows other granule cells undergoing degeneration where fragmentation of the nucleus and vacuolation in the cytoplasm is very apparent (black arrow). Scale bar = 5 µm. (**B**) High power microscopy of degenerated granule cells in the hippocampus of adrenalectomized rats 14 days following adrenalectomy. Appearance of vacuolation in the cytoplasm with condensed chromatin (thin arrow) and adjacent granule cell in the process to be engulfed by microglia (big arrow). Scale bar = 1 µm. (**C**) High power microscopy taken from CA4 area of the hippocampus following 14 days of adrenalectomy showing many empty spaces in the pyramidal layer. In addition, many neurons with compacted chromatin surrounded by irregular nuclear membrane black arrow. A glial cell in the process to phagocyte pyramidal cell of the CA4 white arrow. Scale bar = 5 µm. (**D**) Electron micrograph showing CA3 pyramidal cells at different stages of degeneration 14 days following adrenalectomy, confirming that withdrawal of glucocorticoids by adrenalectomy is not selective where different neuronal populations in the hippocampus are subjected to substantial degeneration. Scale bar = 2 µm. Three independent sections per brain were examined (*n* = 3 in each group).

We observed, 14 days post-adrenalectomy, an extensive cell death on the tip of the upper blade of the dentate gyrus, followed by a lesser degree of cell death in the lower blade. Through the examination of the granule layer of the dentate gyrus, we observed differences in the gross appearance of the neuropil between the ADX and SHAM operated rats. In the ADX animals, the granule layer showed empty areas with multiple “blank” spaces that indicated already degenerated cells. In addition to the changes in the neuropil, we observed ultrastructural changes in the upper and lower blades of the dentate gyrus (Figure 7A).

We have observed, 14 days following adrenalectomy, the condensation of chromatin beside the shrinkage of the cell body, revealing both apoptotic and necrotic process are occurring in the dentate gyrus of the hippocampus. These observations indicate the major vulnerability of the granule cells of the hippocampus to the absence of glucocorticoids achieved by the removal of the adrenal gland (Figure 7B). In contrast, in the SHAM operated rats the neuropil was continuous with abundant, tightly packed granule cells. As the granule cells are small neurons, the nucleus occupied most of the cell body with only a thin rim of cytoplasm surrounding it.

The examination of the Cornu Ammonis of the ADX rats revealed an extensive cell death in the CA4 area where chromatin condensation and vacuolation were also seen (Figure 7C). Moreover, the electron microscopy examination of the CA3 area of the ADX rats, 14 days post adrenalectomy, revealed the occurrence of cell death of the pyramidal cells along the principal layer of this area where extensive chromatin condensation, reduction in soma volume, wavy shrinkage of the nuclear membrane, and the vacuolization of mitochondria all occur (Figure 7D). SHAM operated rats exhibited healthy clustered pyramidal cells with a regular plasma membrane, and a well-defined nucleus with consistent dispersed chromatin. More importantly, our results showed no ultrastructural abnormalities in the CA1 and CA2 pyramidal cells of both groups (results not shown).

### 3.4. Growth Factors Analysis

**Figure 8:**
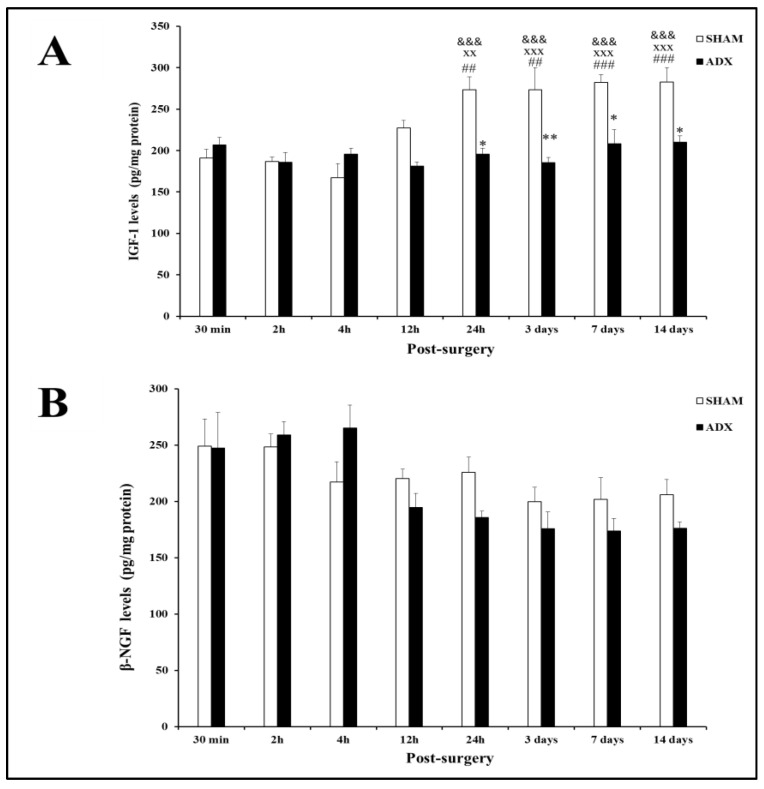
Bar graph showing IGF-1 (**A**) and β-NGF (**B**) levels in the hippocampus of ADX rats and SHAM operated rats. IGF-1 levels were measured by ELISA over the course of time (0.5, 2, 4, 12 h, and 1, 3, 7, 14 days). Data are expressed as mean (±SEM). SHAM versus 24 h, 3 days, 7 days and 14 days ADX (* *p* < 0.05; ** *p* < 0.01). 30 min-SHAM versus (24 h, 3 days, 7 days, 14 days) SHAM groups (^##^ *p* < 0.01; ^###^ *p* < 0.001). 2 h-SHAM versus (24 h, 3 days, 7 days, 14 days) SHAM groups (^XX^ *p* < 0.01; ^XXX^ *p* < 0.001). 4 h-SHAM versus (24 h, 3 days, 7 days, 14 days) SHAM groups (^&&&^ *p* < 0.001).

Using ELISA, our results showed differences in IGF-1 protein levels. In the hippocampus, the IGF-1 protein levels were significantly decreased in the ADX rats at 24 h (*p* < 0.05) and remained consistently low at 3 days (*p* < 0.01), 7 days (*p* < 0.05), and 14 days (*p* < 0.05) in the hippocampus of ADX rats compared to the SHAM operated rats (Figure 8A). Moreover, our results showed a significant increase in the levels of IGF-1 with time in the SHAM groups, which peaked after 24 h, and continued at the same level for the rest of the experiment (3, 7, and 14 days).

The evaluation of the levels of β-NGF in the hippocampus homogenates of ADX rats did not exhibit any changes compared to the SHAM operated rats in the different time points post adrenalectomy (Figure 8B). However, we observed a significant decrease in the levels of β-NGF in ADX rats three days post-surgery, and they remained at this level for the rest of the experiment (3, 7 and 14 days).

### 3.5. Animal Behavior Test

**Figure 9:**
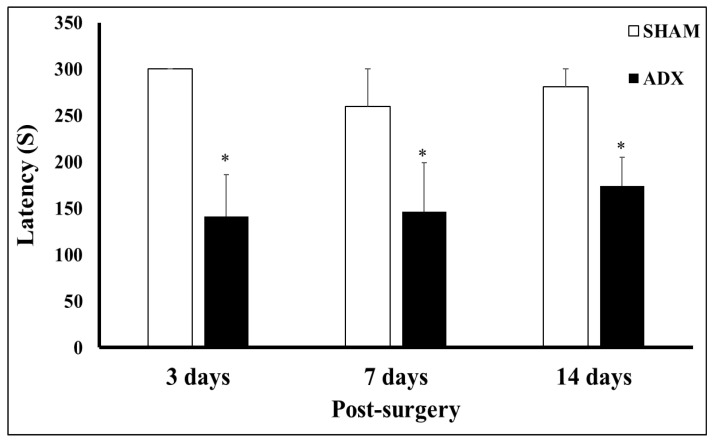
Bar graphs showing latency time on the retention day of ADX and SHAM operated rats following 3, 7, and 14 days following bilateral adrenalectomy. * *p* < 0.05. 3 days (SHAM = 6, ADX = 9), 7 days (SHAM = 8, ADX = 10), and 14 days (SHAM = 7, ADX = 16).

Based on our histological and biochemical findings in the hippocampus, we expected a negative impact on the cognitive behavior of the ADX rats, and hence the Passive Avoidance Task (PAT) was performed. The evaluation of the latency time in the Passive Avoidance Task at different time points (3, 7, and 14 days) indicated a significant decrease in the ADX compared to SHAM operated rats, on the third (*p* < 0.05), seventh (*p* < 0.05), and fourteenth day (*p* < 0.05) following surgery (Figure 9).

## 4. Discussion

In our previous studies, we reported that the absence of glucocorticoids for a long period of time (5 months) induces non-selective degeneration in the hippocampus, where different neuronal populations are affected, and exhibit signs of abnormalities and different cell death types [7,8]. Therefore, in the present study using stereological techniques and ultrastructural examination, we aimed to investigate the impact of short-term adrenalectomy (2 weeks) on different hippocampal neuronal populations in Wistar rats. In addition, the levels of two important growth factors in the hippocampus, insulin-like growth factor-1 (IGF-1) and β-nerve growth factor (β-NGF), were investigated. Moreover, we examined whether the biochemical and histological changes in the hippocampus, after short-term adrenalectomy, have an impact on the cognitive behavior of Wistar rats.

Our results showed, half an hour following the bilateral removal of the adrenal glands, a drastic depletion in the levels of corticosterone in the ADX, compared to the SHAM operated rats. Such a decrease persists over the course of time of the experiment (0.5, 2, 4, 12 h, and 1, 3, 7, 14 days). The adrenal gland is the major producer of corticosterone in the body, and the significant depletion of corticosterone that was observed in the ADX rats is attributed to its removal [1,3,38,39,40,41].

The impact of long-term removal of the adrenal gland on neurodegeneration of hippocampal neurons has been the subject of extensive research. However, there is controversy as to whether long-term removal selectively affects the granule cells or not. Long-term withdrawal of the glucocorticoids by adrenalectomy was wrongly suggested as a selective model of granule cells’ degeneration [1,2,3,4]. However, Sapolsky et al. [6] and Adem et al. [7] showed that, indeed, the pyramidal cells are also vulnerable to such hormonal manipulation.

We have previously shown, using Fluoro-Jade B (FJB) staining, a few dentate granule cells’ deaths on day three, which increased progressively on day 7 and 14 post adrenalectomy [42]. In line with our previous findings, in the present study, using a stereological counting technique, we observed a decrease in the number of granule cells at day 3— albeit not significant—which further decreased, significantly, at day 7 and 14 post adrenalectomy. In addition, the stereological counting revealed a significant decrease in the number of CA4/CA3 pyramidal cells at day 7 and 14 post adrenalectomy.

Our ultrastructural observation of the hippocampus using transmission electron microscopy showed that granule cells of the dorsal blade appeared to be the first cells that were affected by the absence of glucocorticoids, which occurred on the third day following adrenalectomy. Our results showed cell abnormalities appeared in the form of condensed chromatin, and an irregular cell membrane. These observations are in line with the findings of Sloviter et al. [3,4] where they examined the granule cells’ degeneration after four days following adrenalectomy. In contrast to the granule cells, no significant signs of damage have been seen along the pyramidal cells of the Cornu Ammonis at this time point.

Interestingly, seven days post adrenalectomy, we observed the cell death of CA4 and CA3 pyramidal cells, in addition to significant granule cell degeneration in both blades of the dentate gyrus. Moreover, 14 days post adrenalectomy, a substantial cell loss was observed all over the dentate gyrus, where the morphological abnormalities included cell membrane disintegration and chromatin condensation, as well as the invasion of the damaged area by glia. In addition, we observed a progression in pyramidal cells’ degeneration of CA4 and CA3 where more damaged and affected cells were seen. Similarly to the granule cells, the pyramidal cells showed morphological abnormalities, such as cell membrane disintegration, chromatin condensation, and invasion of the damaged area by glia.

Few studies have investigated the relationship between glucocorticoids and IGF-1. Interestingly, the levels of IGF-1 in the hippocampus of the SHAM operated rats were very low in the early hours after adrenalectomy when the levels of corticosterone were high (trauma of surgery). However, when the levels of corticosterone returned to normal (24 h, 3, 7, and 14 days) the IGF-1 levels were significantly higher than the levels of IGF-1 at 30 min, 2 h, and 4 h after adrenalectomy. These findings suggest that the levels of IGF-1 in the hippocampus are low when the corticosterone level is high, and vice versa.

Dexamethasone was shown to down regulate IGF-1 mRNA levels in rat neuronal and glial cells in vitro [28]. Downregulation of gene transcription by glucocorticoids has also been observed in the IGF-1 gene. Cortisol decreased the transcriptional activity of the IGF-1 gene in human osteoblast cells in vitro [29]. Recently, it has been demonstrated that postnatal injection of dexamethasone causes a significant decrease in the levels of IGF-1, accompanied by cell death in the hippocampus [43]. In line with the latter, it has been shown that in Alzheimer’s disease, the cortisol levels are significantly elevated with low levels of IGF-1 [44]. Although it has been shown that high levels of cortisol or dexamethasone significantly decrease IGF-1 levels, there are no reports as to the effect of low cortisol on IGF-1 levels. In the current study, after short-term bilateral adrenalectomy, we observed a significant reduction in IGF-1 levels in the hippocampus at 24 h, 3 days, 7 days, and 14 days. These findings suggest that a lack of glucocorticoids leads to significant decreases in IGF-1 levels in the hippocampus.

Numerous studies have shown that IGF-1 deficiency, specifically in the brain, results in various abnormalities, including neurodegeneration. IGF-1 deficiency leads to increased oxidative damage to the brain, causes edema, and impairment in learning and memory processes, which can be restored with IGF-1 replacement therapy [45]. A systematic anatomical evaluation of the brains of IGF-1 -/- mice revealed decreased brain size, hypomyelination of the central nervous system, and loss of neurons from the hippocampus and striatum [46]. Furthermore, astrocytic infiltration is another abnormal function associated with IGF-1 scarcity in the brain [47]. It could be suggested that reduced IGF-1 levels in the brain cause IGF-1 receptor downregulation, leading to dysregulation of the several secondary messenger pathways, including PI3K/Akt/mTOR and MAPK/ERK1/2, which might result in neuronal degeneration, apoptosis, and a decline in cognitive function. 

Our present results support our previous findings [48] where we observed an alteration of IGF-1 receptor and its messenger ribonucleic acid (mRNA) in the hippocampus after long-term adrenalectomy, using in vitro receptor autoradiography and in situ hybridization immunohistochemistry, respectively. Significantly decreased levels of IGF-1 receptor and its mRNA were noted in the DG and CA1–CA4 regions of the hippocampus after long-term adrenalectomy, suggesting that the level and expression of IGF-1 receptors in the hippocampus is influenced by adrenal hormones [48]. In the present study, the significant reduction in IGF-1 seen at 24 h after adrenalectomy takes place long before the death of the neurons, which is observed on day three post adrenalectomy. Our results suggest that bilateral adrenalectomy increases the susceptibility of hippocampal neurons’ degeneration through an early decrease in IGF-1 levels. These findings indicate that IGF-1 may exert potent neurotrophic and neuroprotective/antiapoptotic activities in the hippocampus, as suggested previously [11,49,50].

In contrast to IGF-1, the level of β-NGF was not significantly reduced in the hippocampus after short term adrenalectomy. Although there was a trend of decreasing levels of ADX β-NGF compared to SHAM, starting from 12 h to 14 days, it did not reach statistical significance. However, the levels of ADX β-NGF at 2 h and 4 h were significantly higher than those at 3, 7, and 14 days. In the early hours (30 min, 2 h, and 4 h) after adrenalectomy (trauma of surgery), the level of corticosterone in the ADX rats was higher than during 3, 7, and 14 days after adrenalectomy. These findings suggest that, in contrast to IGF-1, β-NGF levels increase and/or decrease with the levels of corticosterone. However, our findings are not in line with those of Aloe [26] who found a significant decrease in the levels of β-NGF in the young rat hippocampus 12 days after adrenalectomy. Taken together, these findings indicate that β-NGF might not be involved in the early neurodegeneration seen in the hippocampus after bilateral adrenalectomy. In addition to the early decrease in IGF-1, other trophic factors such as β-NGF, which have impacts on hippocampal neurons, might be affected later after bilateral adrenalectomy, and may exacerbate the neurodegenerative process in the hippocampus.

The hippocampus is well known for its role in cognition [51,52], most importantly in learning and memory [53]. As it was aforementioned, due to the high concentration of glucocorticoid receptors in the hippocampus, it is considered the area of the brain most targeted by adrenal hormones [54,55]. Several studies have shown the withdrawal of these hormones causes degeneration of different neuronal cell populations in the hippocampus. The impact of the neuronal damage caused by long-term absence of adrenal hormones on the behavior of rats was examined by different investigators. We showed, 19 weeks post adrenalectomy, a significant increase in the latency in the Morris maze task, and significantly lower rearing scores from the ADX rats compared to the SHAM operated rats [56]. A significant decrease in ambulatory and rearing activities was observed following chronic adrenalectomy, while grooming and defecation scores were not altered [57]. Moreover, Conrad and Roy [58] showed that after long-term adrenalectomy, the ADX rats exhibited difficulties in acquiring new spatial memory. Furthermore, Spanswick et al. [31] showed the inability to reverse such deficit in the spatial memory of long-term ADX rats by chronic treatment (6 weeks) with corticosterone, or by an alternative neurogenic compound, fluoxetine. The latter finding indicates the crucial role of hippocampal formation in building memories, and acquiring new information. In the current study, using the passive avoidance task, we investigated the effect of short-term adrenalectomy on cognitive functions. Our results showed that the ADX rats failed to retain their cognitive capacity compared to the SHAM operated rats, as we have seen on days 3, 7, and 14 a significant decrease in the latency of the ADX rats, revealing a cognitive decline in these rats. The cognitive decline seen on day three after adrenalectomy (despite no significant cell death) could possibly be due to the early (24 h) significant decreases in IGF-1 levels. Moreover, the significant low levels of IGF-1 might also have an impact on the cognitive decline seen at 7 and 14 days. Our present findings are in line with those of Oitzl and de Kloet [59] who found that adrenalectomy impaired the performance of rats in the maze 3 days after the surgery. Moreover, it was reported that ADX rats were also significantly less active than controls on days 3 and 4 of testing, as a result of a greater drop in activity over the four days of testing [60]. Taken together, our present results and those of others [56,58,59,60] are in line with our previous results of long-term adrenalectomy, and suggest that cognitive behavior impairment might start as early as day 3 following glucocorticoid withdrawal.

## 5. Conclusions

We have previously showed that short-term (two weeks) bilateral adrenalectomy leads to an early increase in proinflammatory cytokines, followed by neurodegeneration and activation of glial cells, as well as oxidative stress [42]. In the present study, the significant reduction in IGF-1 in the hippocampus, seen at 24 h after adrenalectomy, takes place long before the death of the neurons which is observed on day three post adrenalectomy. Our results suggest that bilateral adrenalectomy increases the susceptibility of hippocampal neurons to degeneration through an early decrease in IGF-1 levels. Taking together our previous and present findings, it could be suggested that in addition to the early inflammatory components, an early IGF-1 loss might contribute to the initiation of the biological cascade responsible for subsequent hippocampal neuronal cell death in the current neurodegenerative animal model. These findings indicate that IGF-1 plays an important role as a neurotrophic factor for hippocampal neurons. Moreover, IGF-1 might have a role in the early cognitive decline after adrenalectomy.

In conclusion, our results showed hippocampal granule and pyramidal neuron degeneration after short-term adrenalectomy. Consequently, the loss of the hippocampal neurons after adrenalectomy led to cognitive behavioral deficits.

## Abbreviations

ADX	Adrenalectomy
ANOVA	Analysis of Variance
β-NGF	β-Nerve Growth Factor
CA areas	Cornu Ammonis
CAST	Assisted Stereological Toolbox
CORT	Corticosterone
CNS	Central Nervous System
DG	Dentate Gyrus
EIA	Enzyme Immunoassay
ELISA	Enzyme-Linked Immunosorbent Assay
FJB	Fluoro-Jade B
IGF-1	Insulin-Like Growth Factor-1
mRNA	Ribonucleic Acid
PA	Passive Avoidance
SRS	Systematic Random Sampling
